# Targeting Oxidative Phosphorylation with a Novel Thiophene Carboxamide Increases the Efficacy of Imatinib against Leukemic Stem Cells in Chronic Myeloid Leukemia

**DOI:** 10.3390/ijms252011093

**Published:** 2024-10-15

**Authors:** Kana Kusaba, Tatsuro Watanabe, Keisuke Kidoguchi, Yuta Yamamoto, Ayaka Tomoda, Toshimi Hoshiko, Naoto Kojima, Susumu Nakata, Shinya Kimura

**Affiliations:** 1 Division of Hematology, Respiratory Medicine and Oncology, Department of Internal Medicine, Faculty of Medicine, Saga University, Saga 849-8501, Japan; sp7378@cc.saga-u.ac.jp (K.K.); st9179@cc.saga-u.ac.jp (K.K.); 2 Department of Drug Discovery and Biomedical Sciences, Faculty of Medicine, Saga University, Saga 849-8501, Japan; sn6538@cc.saga-u.ac.jp (T.W.); yuyama@cc.saga-u.ac.jp (Y.Y.); sj7976@cc.saga-u.ac.jp (A.T.); 3 Clinical Research Center, Saga Medical School Hospital, Saga 849-8501, Japan; sy5886@cc.saga-u.ac.jp; 4 Faculty of Pharmaceutical Sciences, Nagasaki International University, Nagasaki 859-3298, Japan; kojima@niu.ac.jp; 5 Laboratory of Clinical Oncology, Kyoto Pharmaceutical University, Kyoto 607-8414, Japan; snakata@mb.kyoto-phu.ac.jp

**Keywords:** oxidative phosphorylation, thiophene carboxamide, tyrosine kinase, chronic myeloid leukemia, stem cell

## Abstract

Patients with chronic myeloid leukemia (CML) respond to tyrosine kinase inhibitors (TKIs); however, CML leukemic stem cells (LSCs) exhibit BCR::ABL kinase-independent growth and are insensitive to TKIs, leading to disease relapse. To prevent this, new therapies targeting CML-LSCs are needed. Rates of mitochondria-mediated oxidative phosphorylation (OXPHOS) in CD34^+^CML cells within the primitive CML cell population are higher than those in normal undifferentiated hematopoietic cells; therefore, the inhibition of OXPHOS in CML-LSCs may be a potential cure for CML. NK-128 (C_33_H_61_NO_5_S) is a structurally simplified analog of JCI-20679, the design of which was based on annonaceous acetogenins. NK-128 exhibits antitumor activity against glioblastoma and human colon cancer cells by inhibiting OXPHOS and activating AMP-activated protein kinase (AMPK). Here, we demonstrate that NK-128 effectively suppresses the growth of CML cell lines and that the combination of imatinib and NK-128 is more potent than either alone in a CML xenograft mouse model. We also found that NK-128 inhibits colony formation by CD34^+^ CML cells isolated from the bone marrow of untreated CML patients. Taken together, these findings suggest that targeting OXPHOS is a beneficial approach to eliminating CML-LSCs, and may improve the treatment of CML.

## 1. Introduction

Chronic myeloid leukemia (CML) is a clonal myeloproliferative neoplasm characterized by the unregulated expansion of myeloid cells in the bone marrow [1]. Most patients with CML-CP (chronic phase) respond well to tyrosine kinase inhibitors (TKIs), and approximately one-third of them can safely discontinue TKIs after achieving a sufficiently deep molecular response (DMR) [2]; however, some patients harbor residual leukemia cells. This means that disease recurrence usually occurs after TKI discontinuation because surviving CML leukemic stem cells (LSCs) are BCR::ABL kinase-independent and insensitive to TKIs [3,4]. Cancer stem cells are poorly differentiated or quiescent cells whose presence is associated with drug resistance and tumor relapse [5]. In addition, metabolic pathways utilized by cancer stem cells differ from those used by normal cells. To prevent relapse, new therapies that selectively target CML-LSCs by focusing on these different metabolic pathways are needed. Studies show that levels of mitochondria-mediated oxidative phosphorylation (OXPHOS) are greater in CD34^+^ CML cells, and that mitochondrial metabolic activity in primitive CML cells is higher than in normal undifferentiated hematopoietic cells [5,6]. This suggests that inhibiting mitochondrial OXPHOS in CML-LSCs may be a potential cure for CML.

**Figure 1:**
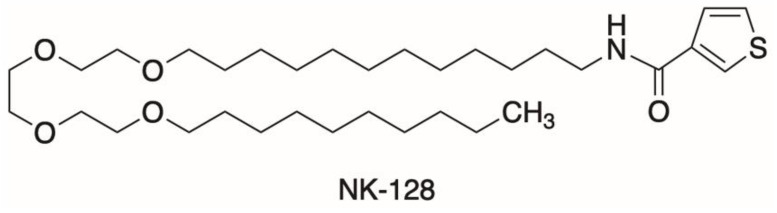
Chemical structure of thiophene carboxamide (NK-128).

NK-128, characterized by a thiophene carboxamide with a long alkyl chain that includes a triethylene glycol moiety, is a structurally simplified analog of JCI-20679. The design of JCI-20679 is based on annonaceous acetogenins, a type of polyketide isolated from Annona plants (Figure 1) [7]. JCI-20679 exhibits potent antitumor activity against human lung cancer NCI-H23 cells [8,9] and glioblastoma [10,11]. Additionally, NK-128 exerts antitumor activity against glioblastoma and human colon cancer SW48 cells, despite being synthesized in only five steps from commercially available compounds (the synthesis of JCI-20679 requires 23 steps). Furthermore, NK-128 activates AMP-activated protein kinase (AMPK) by inhibiting OXPHOS in cancer cells. 

Therefore, we hypothesized that NK-128 could eliminate OXPHOS-dependent CML-LSC and that the combination of NK-128 with a TKI would enhance this effect. In the present study, we report that NK-128 effectively suppressed the growth of CML cell lines by inhibiting proliferation and that the combination of the TKI imatinib and NK-128 was more potent than either agent alone in a xenograft CML mouse model. Furthermore, we found that NK-128 exerted significant growth-inhibitory activity against human CML progenitor and stem cells isolated from the bone marrow of untreated CML patients.

## 2. Results

### 2.1. NK-128 Inhibits the Growth of CML and Ph^+^ ALL Cell Lines

**Figure 2:**
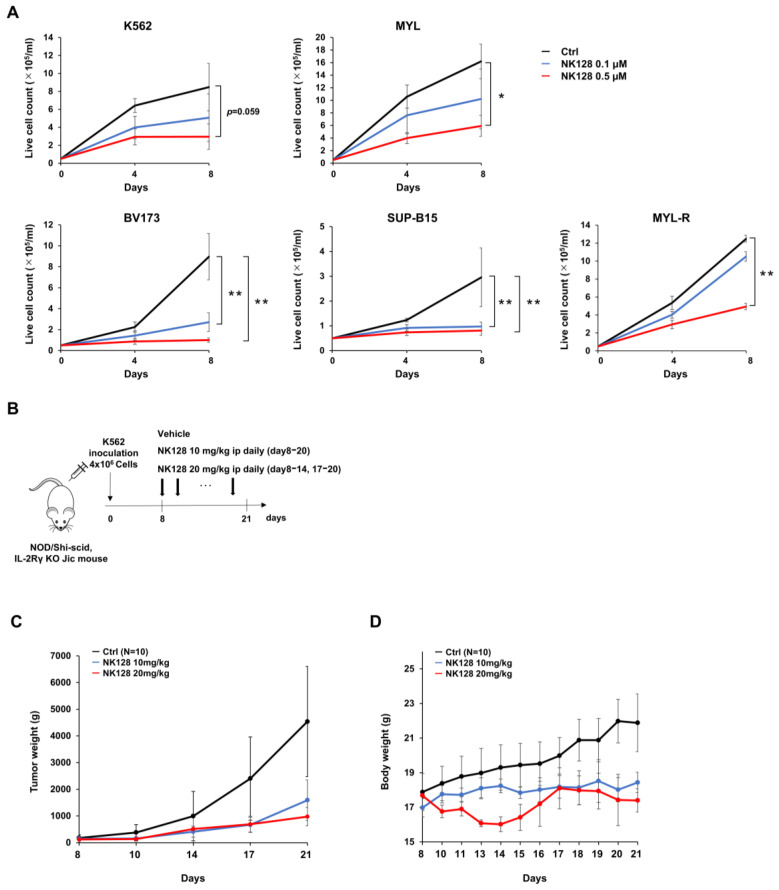
NK-128 inhibits the proliferation of CML and Philadelphia chromosome-positive acute lymphoblastic leukemia cell lines. (**A**) Cells were incubated with NK-128 and cell numbers were counted on Days 4 and 8. The number of viable cells is shown. NK-128 inhibited the proliferation of BV173 (*p* < 0.01) and SUP-B15 (*p* < 0.05) cells significantly, even at concentrations as low as 0.1 μM. (* *p* < 0.05, ** *p* < 0.01). (**B**) Experimental scheme showing the generation of the K562 xenograft model using NOD/Shi-scid IL-2Rγ KO Jic mice. On Day 0, K562 cells (4.0 × 10^6^ cells/mouse) were injected subcutaneously into NOD/Shi-scid IL-2Rγ KO Jic mice. Daily ip administration of the vehicle and NK-128 began on Day 8. (**C**) Tumor growth curves and (**D**) body weight for each group; vehicle (black; n = 10), 10 mg/kg NK-128 (blue; n = 10), and 20 mg/kg NK-128 (red; n = 10).

First, we analyzed the growth-inhibitory effects of NK-128 on CML and Ph^+^ ALL cells. NK-128 inhibited the proliferation of leukemia cells in a concentration-dependent manner (Figure 2A); BV173 and SUP-B15 cells tended to be more sensitive than K562, MYL, and MYL-R (an imatinib-resistant subline of MYL). BV173 and SUP-B15, the growth of which was inhibited by 0.1 μM NK128, were defined as NK128-sensitive cell lines; however, higher concentrations of NK-128 did not increase the number of dead cells (Supplementary Figure S1A). These results indicate that NK-128 mainly inhibits cell proliferation rather than inducing cell death. Treatment with NK-128 significantly suppressed tumor cell growth in a xenograft mouse model without severe side effects (Figure 2B–D).

### 2.2. The Combination of NK-128 Plus Imatinib Shows Increased Efficacy against CML and Ph^+^ ALL Cell Lines

**Figure 3:**
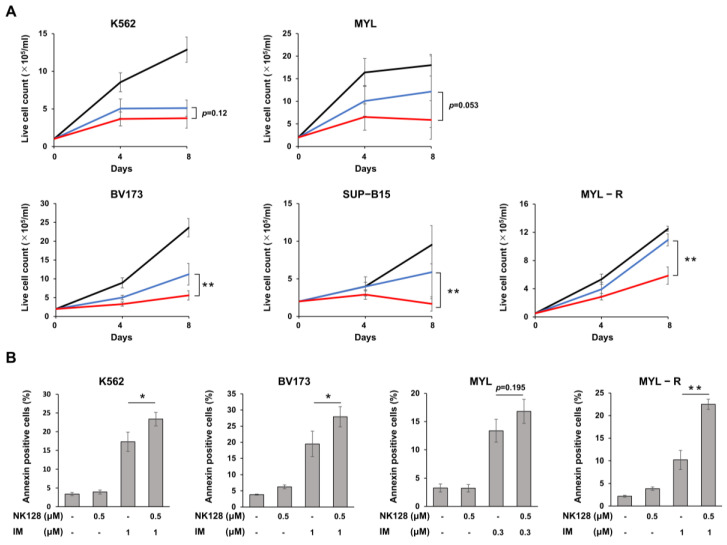
Combination therapy with imatinib and NK-128 inhibits the proliferation of CML cell lines and Philadelphia chromosome-positive acute lymphoblastic leukemia cell lines to a greater extent than imatinib alone. (**A**) CML and Ph^+^ALL cell lines were treated with medium (black), imatinib (blue) or a combination of imatinib and 0.1 μM NK-128 (red). The concentration of imatinib was 0.25 μM for K562, 0.2 μM for MYL, 0.1 μM for BV173, 0.4 μM for SUP-B15, and 1 μM for MYL-R. The combination of NK-128 plus imatinib also inhibited the proliferation to a greater extent than imatinib alone. (** *p* < 0.01) (**B**) Each cell line was treated with or without NK-128 and imatinib (IM) for 3 days. The number of cells stained with APC-annexin V was measured by flow cytometric analysis, as described in the Section 4. Results are the mean of three independent experiments with SD (* *p* < 0.05, ** *p* < 0.01).

Next, we analyzed the inhibitory effects of imatinib combined with NK-128. The combination of the IC50 dose of imatinib (as shown in Supplementary Figure S2) with 0.1 μM NK-128 inhibited the proliferation of each cell line to a greater extent than either agent alone (Figure 3A); in BV173 and SUP-B15 cells, combination treatment was more effective (*p* < 0.01) than imatinib alone, and the effect was more pronounced than that observed for the other cell lines. These results suggest that a combination of imatinib plus NK-128 shows enhanced antitumor effects. Again, the combination of 0.1 μM NK-128 with imatinib did not induce significant cell death (Supplementary Figure S1B); whereas, a higher dose (0.5 μM) of NK-128 enhanced imatinib-induced apoptosis compared to imatinib monotherapy (Figure 3B).

### 2.3. The Growth-Inhibitory Effects of NK-128 in CML Cell Lines Might Be Dependent on Either Glycolysis or OXPHOS

**Figure 4:**
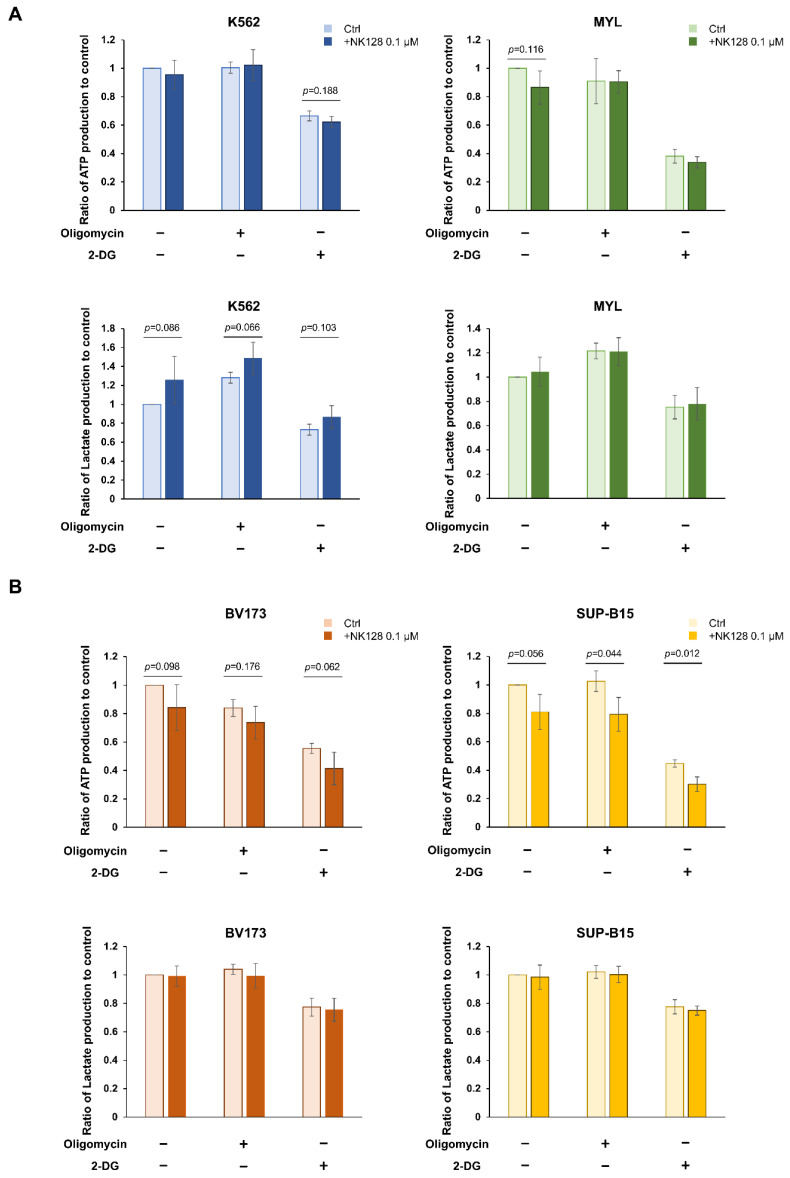
ATP and lactate production by K562, BV173, MYL, and SUP-B15 cells. Oligomycin inhibits OXPHOS, and 2-DG inhibits glycolysis. ATP and lactate production levels were determined in K562 and MYL cell lines, which are less sensitive to NK-128 (**A**) or in the sensitive cell lines, BV173 and SUP-B15 (**B**), as described in the Section 4. The main metabolic pathways of K562 and MYL are glycolysis-dependent. Conversely, those of BV173 and SUP-B15 are OXPHOS-dependent. NK-128 inhibits the production of ATP in BV173 and SUP-B15 cells, which are mainly OXPHOS dependent.

When oligomycin was administered to K562 and MYL cells, which were relatively less sensitive to NK-128 (Figure 2), the inhibition of OXPHOS resulted in minimal changes in ATP levels, while lactate production increased (Figure 4A). This observation is consistent with the well-established phenomenon that activation of glycolysis converts glucose into lactate, thereby increasing lactate production. These findings suggested that K562 and MYL cells, which exhibit lower sensitivity to NK-128, primarily relied on glycolysis for their energy needs. In contrast, oligomycin treatment of BV173 and SUP-B15 cells, which were highly sensitive to NK-128 (Figure 2), led to a significant decrease in ATP levels without a corresponding increase in lactate production (Figure 4B). Given that inhibiting OXPHOS reduces ATP synthesis, these results indicated that BV173 and SUP-B15 cells predominantly depended on OXPHOS for energy production. Overall, these findings imply that NK-128 may exert a more potent inhibitory effect on cells that rely heavily on OXPHOS for energy metabolism. We also confirmed that the combination of NK-128 with oligomycin inhibited cell growth compared to either monotherapy (Supplementary Figure S3).

### 2.4. Combined Treatment with NK-128 and Imatinib Shows Stronger Antitumor Effects in a CML Xenograft Mouse Model

**Figure 5:**
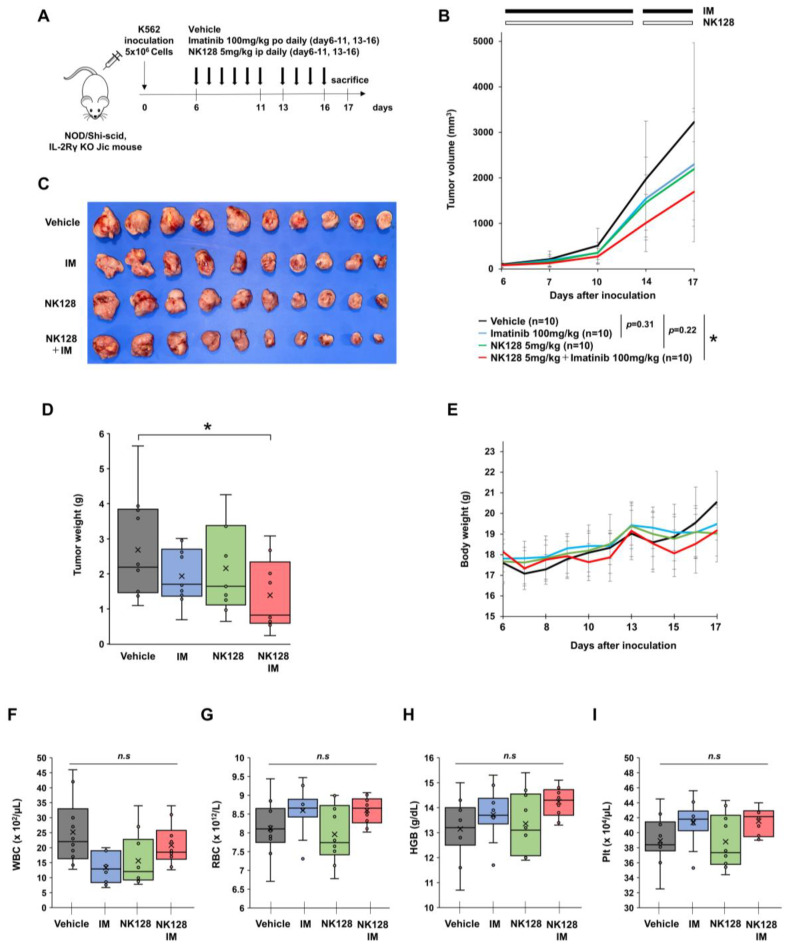
Combined treatment with NK-128 and TKI effectively suppresses tumor growth in xenograft model mice. (**A**) Experimental scheme showing the generation of the K562 xenograft model using NOD/Shi-scid IL-2Rγ KO Jic mice. On Day 0, K562 cells (5.0 × 10^6^ cells/mouse) were injected subcutaneously into NOD/Shi-scid IL-2Rγ KO Jic mice. Vehicle, imatinib, and NK-128 were administered on Day 6 and continued daily for a total of 11 days (nothing was administered on Day 12). (**B**) The number of subjects in each group was determined by the following formula. (**B**) Tumor growth curves for each group. Vehicle (black; n = 10), imatinib (blue; n = 10), NK-128 (green; n = 10), and NK-128 + imatinib (red; n = 10). NK-128 + imatinib inhibited tumor growth significantly (versus vehicle: * *p* = 0.03). (**C**) Xenograft tumors were isolated on day 17 in each treatment group. They were arranged in descending order from the right side. (**D**) The weight of the isolated tumors was measured. NK-128 + imatinib inhibited xenograft tumor growth to a greater extent than vehicle (* *p* = 0.04) Each circle shows the sample value and the cross shows the median. (**E**) Bodyweight was measured from day 6 to day 17. (**F**) White blood cell count, (**G**) red blood cell count, (**H**) hemoglobin levels, and (**I**) platelet count after treatment. Differences between the vehicle and each treatment group were tested using Dunnett’s test. (* *p* < 0.05. n.s. not significant).

Next, we evaluated the antitumor effects of NK-128 plus imatinib in vivo. To do this, we used mice bearing human K562 CML xenografts (Figure 5A). Combination therapy with NK-128 (5 mg/kg) and imatinib (100 mg/kg), which are low doses that have no significant effect when used as single agents, suppressed tumor growth, tumor size, and tumor weight significantly when compared with controls (Figure 5B–D). These results indicate that the combination of NK-128 and imatinib shows stronger antitumor effects not only in vitro but also in vivo. 

In addition, we noted no significant reduction in body weight post-treatment (Figure 5E). Furthermore, there were no significant changes in white blood cell counts, red blood cell counts, hemoglobin levels, and platelet counts (Figure 5F–I). These results indicate that the combination of NK-128 and imatinib is safe (at least at the doses used in these experiments).

### 2.5. Combination of NK-128 and Imatinib Efficiently Inhibits Colony Formation by Primary CML Progenitor and Stem Cells

**Figure 6:**
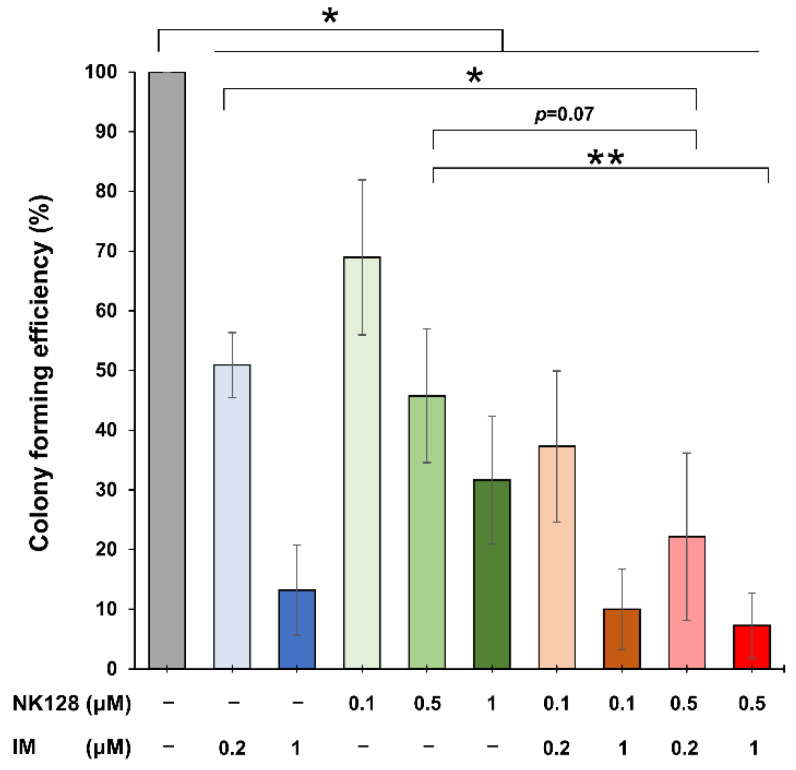
Colony formation by CML CD34+ cells in the presence of different concentrations of imatinib and NK-128. NK-128 monotherapy inhibited colony formation in a concentration-dependent manner (compared with no treatment). NK-128 (500 nM) combined with imatinib (200 nM) inhibited colony formation by CML CD34+ cells significantly compared with imatinib alone. (* *p* < 0.05, ** *p* < 0.01).

Finally, to further analyze the antitumor effects of NK-128 on clinical samples, we examined the effects on colony-forming by CD34^+^ cells isolated from the bone marrow of untreated CML patients. The results showed that compared with the untreated group, imatinib monotherapy or NK-128 monotherapy inhibited colony formation by CML progenitor cells and stem cells (Figure 6). In addition, NK-128 monotherapy significantly reduced leukemia penetrance in secondary HCT mice in the limiting dilution assay, whereas treatment with imatinib did not (Supplementary Figure S4A,B). The combination therapy had an even greater inhibitory effect. In particular, imatinib (200 nM)/NK-128 (500 nM) led to significantly greater inhibition of colony formation than imatinib (200 nM) alone (*p* = 0.028) (Figure 6). 

These results suggest that NK-128 exerts antitumor effects not only against differentiated cells but also against CML cell progenitors and stem cells and is able to inhibit the proliferation of imatinib-resistant CML cell progenitors and stem cells.

## 3. Discussion

Although BCR::ABL inhibitors have improved the prognosis of CML, relapse due to the regrowth of treatment-resistant CML stem cells remains a major clinical problem. Previous studies show that energy metabolism in these therapy-resistant CML stem cells is dependent on activated OXPHOS in the mitochondria [6]. Here, we show that a newly synthesized mitochondrial complex I inhibitor, NK-128 (Figure 1), inhibits the proliferation of leukemia cell lines in vitro (Figure 2) and in vivo (Figure 5). NK-128 showed even stronger growth-inhibitory effects against CML cell lines when used in combination with a TKI. These enhanced antiproliferative effects were also demonstrated in SUB-B15 cells, a Ph^+^ALL cell line (Figure 3).

The efficacy of NK-128 varied according to the cell line used in the assay, possibly due to the differing dependency of the cells on glycolysis or OXPHOS. The efficacy of NK-128 is likely to be greater in cells that rely on OXPHOS for energy metabolism. In fact, of the cell lines treated with NK-128, those that are dependent on OXPHOS tended to exhibit lower ATP production than control cells (Figure 4). A previous report shows that it blocking the glycolytic system inhibits the proliferation of K562 cells, a finding consistent with our analysis [12]. Since normal hematopoietic stem cells (HSCs) depend on the glycolytic system for energy metabolism [13] and CML-LSCs depend on OXPHOS [6], the antitumor effect of NK-128 is expected to be stronger against CML-LSCs and may efficiently eradicate them. Another report that used a mouse model of CML pathogenesis showed that TKI treatment initially inhibits glycolysis, glutamine metabolism, and TCA circuits (including OXPHOS) [14]. Our finding regarding combined treatment with NK-128 and imatinib may be due to the fact that OXPHOS was strongly suppressed.

Since previous studies report that AMPK in malignant tumor cells is activated via the AMPK pathway, resulting in an antitumor effect [15,16], it is likely that NK-128 acts via the same mechanism. In CML, the efficacy of NK-128 is likely high because AMPK activation promotes translocation of the BCR::ABL protein to the cytoplasm, followed by autophagic degradation, in CML cells. [17] While activation of the AMPK pathway induces the apoptosis of cancer cells, it can also promote tumorigenesis depending on the level of nutrients within the tumor microenvironment (TME) [18,19,20], as well as adaptation to metabolic stress [21]; in addition, antitumor effects may be elicited through pathways other than AMPK.

We also found that NK-128 inhibited the colony-forming ability of primary untreated human CML progenitor cells and CML-LSCs. Studies suggest that although TKIs can eradicate cells at different stages of differentiation, they are less effective against undifferentiated CML-LSCs (Figure 6) [21,22]. The results of the colony formation assay suggest that the combination of NK-128 with imatinib has the potential to eliminate not only differentiated cells but also CML-LSCs. 

Study limitations include the fact that although we were able to evaluate hematologic toxicity in a CML mouse model, we did not assess blood biochemistry. A previously reported Phase 1 trial of oxidative phosphorylation inhibitors for advanced solid tumors and acute myeloid leukemia revealed adverse events such as elevated blood lactate and neurotoxicity due to OXPHOS inhibition [23]; therefore, the optimal dose of NK-128 needs to be ascertained. 

In summary, we show here that NK-128 exerts antitumor effects in fresh bone marrow samples from CML patients and against CML cell lines. In addition, co-administration of NK-128 and imatinib results in enhanced antitumor effects via inhibition of the colony-forming capacity of LSCs and progenitor cells. Taken together, these results suggest that combined treatment with NK-128 and imatinib is a promising and potentially curative option for CML.

## 4. Materials and Methods

### 4.1. Cell Lines and Cultures

CML cell lines K562 and BV173 were purchased from the Japanese Collection of Research Bioresources Cell Bank (JCRB, Ibaraki, Osaka, Japan) and DSMZ (Braunschweig, Germany), respectively. CML cell line MYL was kindly provided by Dr. H. Tanaka (Hiroshima City Asa Hospital, Hiroshima, Japan). The Philadelphia chromosome-positive (Ph^+^) acute lymphoblastic leukemia (ALL) cell line SUP-B15 was purchased from the American Type Culture Collection (ATCC, Manassas, VA, USA). Cells were maintained in RPMI-1640 medium (FUJIFILM Wako Pure Chemical Corporation, Osaka, Japan) containing 10% fetal bovine serum (Sigma-Aldrich, St. Louis, MO, USA).

### 4.2. Reagents

NK-128 (*N*-(13,16,19,22-tetraoxidedotriacontan-1-yl)-thiophene-3-carboxyamide) was synthesized as previously described [7]. NK-128 was dissolved in dimethyl sulfoxide (DMSO)/ethanol = 1:1 solution and stored at −80 °C. Imatinib (STI571) for use in cell cultures was purchased from Selleck Biotech (Kanagawa, Japan). Imatinib for use in the xenograft model was purchased from Tokyo Chemical Industry (Tokyo, Japan). Imatinib was dissolved in DMSO and stored at −20 °C. ATP production was measured in a CellTiter-Glo^®^ 2.0 Cell Viability Assay (Promega, Madison, WI, USA) and lactate production was measured using Lactate Assay Kit-WST (Dojindo, Kumamoto, Japan). A dead cell removal kit and a CD34 MicroBead Kit were purchased from Miltenyi Biotec (Bergisch Gladbach, Germany). Methocult H4435 Enriched was purchased from STEMCELL Technologies (Vancouver, BC, Canada).

### 4.3. Cell Growth and Viability

NK128 affects mitochondrial oxidative phosphorylation and suppresses ATP production. Since the Cell Counting Kit-8 (CCK-8, Dojindo Molecular Technology, Kumamoto, Japan) assay is based on the measurement of the metabolic activity (dehydrogenase activity with NADH) of living cells, it is not suitable for cells treated with NK128. Therefore, cell growth was evaluated by the trypan blue dye exclusion method. Cell lines (5 × 10^4^ cells/mL) were cultured with NK-128 alone and counted on Days 4 and 8 and then the number of live and dead cells was counted using an automated cell counter (Luna IITM, Logos Biosystems, Anyang-si, Republic of Korea). On Day 4, the medium was exchanged with medium containing the same drug concentration, and the cell number was adjusted to 5 × 10^4^/mL. Cells (1–2 × 10^5^ cells/mL) were used for combination therapy experiments (i.e., imatinib plus NK-128) and counted on Days 4 and 8. The medium was then exchanged as described in the prior experiment. The IC50 for imatinib was determined using a CCK-8 according to the manufacturer’s instructions 72 h after treatment.

### 4.4. Analysis of Cell Apoptosis

Apoptotic cells were detected by staining with APC-conjugated annexin V and propidium iodide 3 days after treatment with the compounds, followed by flow cytometric analysis. 

### 4.5. Xenograft CML Mouse Model

Animal studies were conducted according to animal protocols approved by Saga University (A2021-027-0, 22 November 2023), in accordance with the German Animal Welfare Act. NOD/Shi-scid IL-2Rγ KO Jic (NOG) female mice (6 weeks of age) were purchased from In-Vivo Science Inc. (Kawasaki, Japan). K562 cells (5 × 10^6^ cells/mouse) were subcutaneously inoculated into NOG mice and randomized into four treatment groups when the average tumor volume reached 100 mm^3^: vehicle, imatinib, NK-128, and imatinib plus NK-128. NK-128 was dissolved in DMSO/ethanol (1:1 solution) and then diluted in saline containing 10% Kolliphor EL. Vehicle (DMSO/ethanol = 1:1 solution, diluted in saline containing 10% Kolliphor EL, intraperitoneal injection; saline, oral gavage; both daily), imatinib (100 mg/kg; oral gavage; daily), and NK-128 (5–10 mg/kg; intraperitoneal injection; daily) were administered starting on Day 6 or 8. Tumor volume was measured twice a week (calculated as (short axis)^2^ × (long axis)/2). Complete blood cell counts were measured using an automated blood cell count analyzer (Nihon Kohden, Shinjuku City, Tokyo, Japan).

### 4.6. ATP and Lactate Assay

ATP levels were measured using a CellTiter-Glo^®^ 2.0 Cell Viability Assay Kit (Promega). To measure the amount of ATP generated by glycolysis, 5 × 10^4^ cells were seeded into each well of a black 96-well plate. To normalize the protein levels, the same number of cells were seeded into clear bottom 96-well plates. Cells were then incubated for 5 h in a medium containing 2.5 μM oligomycin to inhibit ATP production via mitochondrial oxidation or 25 mM 2-deoxy-D-glucose (2-DG) to inhibit ATP production via glycolysis. After 5 h, 20 µL of supernatant was collected and stored at −20 °C prior to use in the lactate assay. The remaining cells were used for ATP measurements. The next day, the supernatant was immediately mixed with reagents from the Lactate Assay Kit (Dojindo), incubated for 30 min at 37 °C, and lactate levels were measured in a microplate reader (EnVision, PerkinElmer, Shelton, CT, USA).

### 4.7. Colony Formation Assay of CD34^+^ CML Cells

Frozen bone marrow cells isolated from CML patients at the time of initial diagnosis were used for the colony formation assays. Briefly, dead cells were removed using a dead cell removal kit (Miltenyi Biotech, Bergisch Gladbach, Germany). Then, CD34^+^ cells were isolated by positive selection using a CD34 MicroBead Kit (Miltenyi Biotech). CD34^+^ cells, including progenitor and stem cells, were cultured in Methocult H4435 Enriched Medium in the presence of each drug, and the number of colonies was counted under a microscope (Leica, Wetzlar, Germany).

### 4.8. Statistical Analysis

All data are expressed as the mean ± standard deviation. Significant differences between the two groups were determined by Dunnett’s test. The EZR software package (version 1.61) was used for all statistical analyses [24] (Saitama Medical Center, Jichi Medical University). 

## Data Availability

The original contributions presented in the study are included in the article/Supplementary Materials, further inquiries can be directed to the corresponding author/s.

## Supplementary Materials

The supporting information can be downloaded at: https://www.mdpi.com/article/10.3390/ijms252011093/s1.
