# Supplementary material for: Targeting Oxidative Phosphorylation with a Novel Thiophene Carboxamide Increases the Efficacy of Imatinib against Leukemic Stem Cells in Chronic Myeloid Leukemia
Source: Int J Mol Sci. 2024 Oct 15;25(20):11093. doi: 10.3390/ijms252011093 (PMC11508009; doi:10.3390/ijms252011093)
Supplement: Supplementary file 1 [file ijms-25-11093-s001.zip › ijms-3268969-supplementary.pdf]

Supplementary information

Targeting Oxidative Phosphorylation with a Novel Thiophene Carboxamide Increases the Efficacy of Imatinib against Leukemic Stem Cells in Chronic Myeloid Leukemia

Kana Kusaba [1], Tatsuro Watanabe [2], Keisuke Kidoguchi [1,2], Yuta Yamamoto [2], Ayaka Tomoda [2], Toshimi Hoshiko [3], Naoto Kojima [4], Susumu Nakata [5] and Shinya Kimura [1,2,*]

[1] Division of Hematology, Respiratory Medicine and Oncology, Department of Internal Medicine, Faculty of Medicine, Saga University, Saga 849-8501, Japan
[2] Department of Drug Discovery and Biomedical Sciences, Faculty of Medicine, Saga University, Saga 849-8501, Japan
[3] Clinical Research Center, Saga Medical School Hospital, Saga 849-8501, Japan
[4] Faculty of Pharmaceutical Sciences, Nagasaki International University, Nagasaki 859-3298, Japan
[5] Laboratory of Clinical Oncology, Kyoto Pharmaceutical University, Kyoto 607-8414, Japan
* Correspondence: shkimu@cc.saga-u.ac.jp; Tel.: +81-952-34-2366; Fax: +81-952-2017

Table of contents:

Supplementary methods
Supplementary Figures S1 to S4

Supplementary methods

Determination of the IC50 for imatinib

The IC50 for imatinib was determined using a Cell Counting Kit-8 (CCK-8) (Dojindo Molecular Technology, Kumamoto, Japan) according to the manufacturer's instructions 72 hours after treatment of CML cell lines with imatinib.

Limiting dilution analysis using a primary CML mouse model

Recipient C57BL/6 mice (8 weeks old, female) were lethally irradiated (9.5 Gy), and then a mixture of GFP+ MIG-BCR-ABL1 retrovirally transduced mouse bone marrow (BM) cells (2×10^4 cells/mouse) and whole BM cells (2×10^5 cells/mouse, for radioprotection) were intravenously injected. BM cells were isolated from recipient mice treated daily with vehicle, IM (100 mg/kg, gavage), and NK-128 (10 mg/kg, intraperitoneally) for 10 days and then transplanted into sub-lethally irradiated secondary recipient mice to perform limiting dilution assay; three different numbers of GFP+ cells (2×10^6, 1×10^6, and 5×10^5 cells/mouse) from each group were transplanted. The survival of mice was monitored.

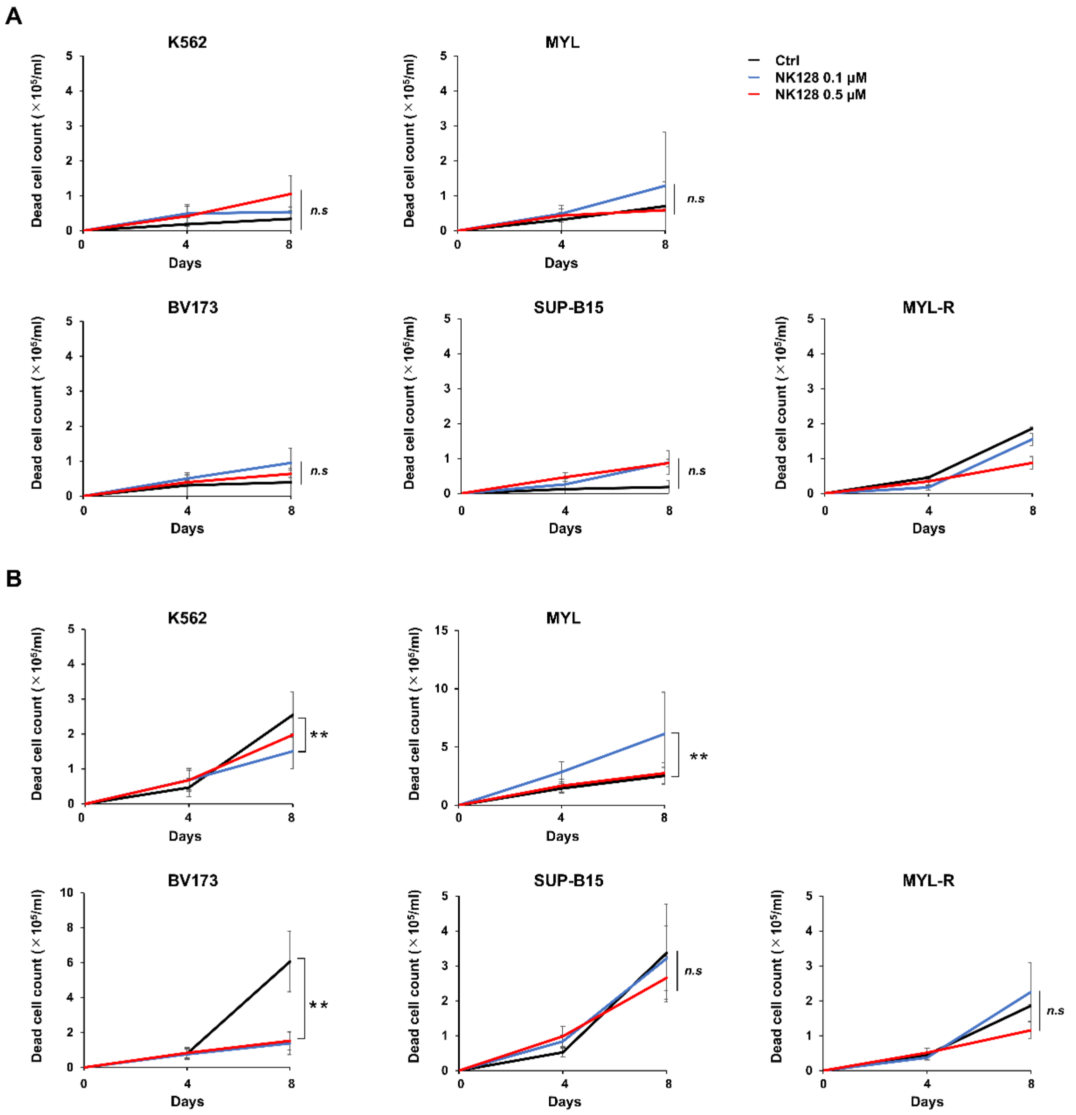

Supplementary Fig. S1. Cancer cell death induced by NK-128 monotherapy and combination therapy with imatinib. A. CML and Ph^+ALL cell lines were treated with NK-128 for 8 days. The number of dead cells was counted by trypan blue staining. **B.** CML and Ph^+ALL cell lines were treated with imatinib (blue) or a combination of imatinib and 0.1 μM NK-128 (red). The concentration of imatinib was 0.25 μM for K562, 0.2 μM for MYL, 0.1 μM for BV173, 0.4 μM for SUP-B15, and 1 μM for MYL-R. The number of dead cells was counted by trypan blue staining. (** $p < 0.01$, n.s. not significant).

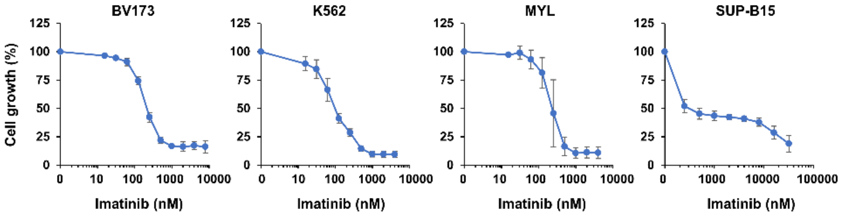

Supplementary Fig. S2. Susceptibility of CML and Ph⁺ALL cell lines to imatinib. CML and Ph⁺ALL cell lines were treated with imatinib for 4 days. Cell growth was accessed using CCK-8 reagent. The absorbance of non-treated cells was defined as 100%. The results are expressed as the mean of three independent experiments ± SD.

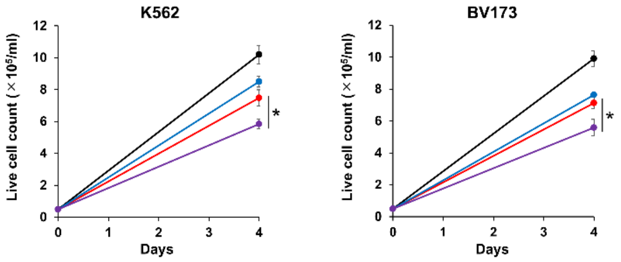

Supplementary Fig. S3. Combination treatment with NK-128 and oligomycin inhibits proliferation of CML cell lines. K562 and BV173 cells were treated with 0.1 μM NK-128 (red), oligomycin (0.25 nM for BV173 and 0.5 nM for K562, blue) or the combination (purple) for 4 days. The number of live cells was counted by trypan blue staining (* $p < 0.05$).

A

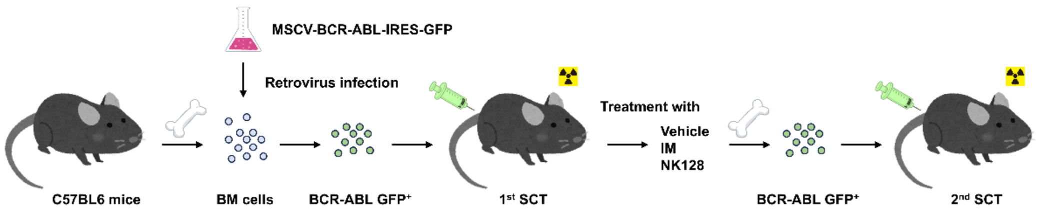

B

Survival at 60 days after 2nd SCT

Group	# of cells	Survival
Vehicle	$1x10^6$	0/4
	$5x10^5$	3/5
IM	$2x10^6$	0/5
	$1x10^6$	0/6
	$5x10^5$	3/5
NK-128	$1x10^6$	5/6
	$5x10^5$	6/6

Supplementary Fig. S4. NK-128 impairs the maintenance of tumor stemness in the CML mouse model. A. Schema of the CML mouse model. Bone marrow (BM) cells transfected with a retroviral vector containing BCR-ABL1 and GFP were transplanted into primary recipient mice. Recipient mice were treated daily with vehicle (1% DMSO, intraperitoneally), NK-128 (10 mg/kg, intraperitoneally), or IM (100 mg/kg, gavage) for 10 days. BM cells were harvested, and transplanted to secondary recipient mice to perform limiting dilution assay; three different numbers of GFP+ cells from each group were transplanted. **B.** Survival of mice at 60 days after secondary transplantation.
